# Risk of developing palatally displaced canines in patients with early detectable dental anomalies: a retrospective cohort study

**DOI:** 10.1590/1678-775720150535

**Published:** 2016

**Authors:** Daniela Gamba GARIB, Melissa LANCIA, Renata Mayumi KATO, Thais Marchini OLIVEIRA, Lucimara Teixeira das NEVES

**Affiliations:** 1 - Universidade de São Paulo, Faculdade de Odontologia de Bauru, Departamento de Odontopediatria, Ortodontia e Saúde Coletiva; Hospital de Reabilitação de Anomalias Craniofaciais, Bauru, SP, Brasil.; 2 - Universidade de São Paulo, Hospital de Reabilitação de Anomalias Craniofaciais, Bauru, SP, Brasil.; 3 - Universidade de São Paulo, Faculdade de Odontologia de Bauru, Departamento de Ciências Biológicas; Hospital de Reabilitação de Anomalias Craniofaciais, Bauru, SP, Brasil.

**Keywords:** Tooth abnormalities, Canine tooth, Etiology, Orthodontics

## Abstract

The early recognition of risk factors for the occurrence of palatally displaced canines (PDC) can increase the possibility of impaction prevention.

**Objective:**

To estimate the risk of PDC occurrence in children with dental anomalies identified early during mixed dentition.

**Material and Methods:**

The sample comprised 730 longitudinal orthodontic records from children (448 females and 282 males) with an initial mean age of 8.3 years (SD=1.36). The dental anomaly group (DA) included 263 records of patients with at least one dental anomaly identified in the initial or middle mixed dentition. The non-dental anomaly group (NDA) was composed of 467 records of patients with no dental anomalies. The occurrence of PDC in both groups was diagnosed using panoramic and periapical radiographs taken in the late mixed dentition or early permanent dentition. The prevalence of PDC in patients with and without early diagnosed dental anomalies was compared using the chi-square test (p<0.01), relative risk assessments (RR), and positive and negative predictive values (PPV and NPV).

**Results:**

PDC frequency was 16.35% and 6.2% in DA and NDA groups, respectively. A statistically significant difference was observed between groups (p<0.01), with greater risk of PDC development in the DA group (RR=2.63). The PPV and NPV was 16% and 93%, respectively. Small maxillary lateral incisors, deciduous molar infraocclusion, and mandibular second premolar distoangulation were associated with PDC.

**Conclusion:**

Children with dental anomalies diagnosed during early mixed dentition have an approximately two and a half fold increased risk of developing PDC during late mixed dentition compared with children without dental anomalies.

## INTRODUCTION

Apart from the third molars, the canines represent the permanent teeth that most commonly show eruptive disorders^14^. The prevalence of cases in which maxillary ectopic canines palatally deviate is 1.7%^14^, commonly affecting three females for each male^24^ ^,^ ^25^ ^,^ ^27^. Less frequently, the maxillary canines are buccally impacted and this irregularity seems to be a clinical manifestation of anterior crowding^20^. The ratio between buccal and palatal impaction of permanent maxillary canines reported in literature is 1:6^20^.

Two theories have been presented to explain the occurrence of palatally displaced maxillary canines (PDC): “guidance” and “genetic” theories. According to the guidance theory, local conditions, such as maxillary lateral incisor agenesis or microdontia, are related to canine displacement^7^ ^,^ ^8^. PDC has a genetic background according to the genetic theory^24^, which was based on observed increased prevalence in families of affected patients, different prevalences between genders and ethnical backgrounds, and increased frequencies of other concomitant dental anomalies^24^ ^,^ ^26^. The search for associated dental anomalies was considered the most relevant method to investigate the genetic determinants of PDC^2^ ^,^ ^24^. Peck, Peck and Kataja^25^ (1996) found that patients with PDC have increased prevalence of permanent tooth agenesis, excluding third molars (17%), and show the mandibular second premolar as the most frequently absent tooth. Additionally, these authors found that approximately 20% of patients with PDC have small lateral incisors not necessarily at the same arch side of the ectopic canine.

The study by Sacerdoti and Baccetti^27^ (2004) does not offer support to the hypothesis that local conditions may be a cause for PDC^7^ ^,^ ^8^, since they did not detect association between the occurrence of bilateral PDC and the occurrence of bilateral agenesis or microdontia of lateral incisors. Additionally, unilateral PDC in cases with unilateral agenesis of maxillary incisors rarely occurs at the same arch side^27^. Sigler, Baccetti and McNamara Jr^30^ (2011) showed that individuals with PDC exhibited significantly higher prevalence of small maxillary lateral incisors (six-fold higher), distoangulation of mandibular second premolars (three-fold higher), and infraocclusion of deciduous molars (two-fold higher) compared with a control group. Other studies verified an increased prevalence of PDC in patients screened for other dental anomalies such as second premolar agenesis, small lateral incisors, infraocclusion of deciduous molars, and enamel hypoplasia^2^ ^,^ ^19^ ^,^ ^29^. Family history has already been identified as a risk factor for PDC and other heritable dental anomalies, as well as the gender bias mentioned^24^ ^,^ ^26^ ^,^ ^27^.

Ectopic eruption of maxillary canines has two major clinical concerns: the consequent impaction of the canine and the possibility of incisor external root resorption^9^ ^,^ ^15^ ^-^ ^18^. The treatment protocol for PDC during permanent dentition is often canine traction, which may present some collateral effects such as root resorption of neighboring teeth, crest bone loss at the mesial aspect of the canine, and tooth discoloration^9^ ^,^ ^13^. Extraction may also be indicated for canines with initial unfavorable position, or in case of tooth ankyloses^16^.

Conversely, when there is an early orthodontic diagnosis of PDC, simpler clinical approaches such as deciduous canine extraction and rapid maxillary expansion can lead to spontaneous canine eruption in a high percentage of children^3^ ^,^ ^5^ ^,^ ^23^ ^,^ ^30^. These early approaches can prevent canine impaction, incisor root resorption, and collateral effects related to tooth traction. Therefore, the recognition of risk factors for the occurrence of PDC can increase the possibility of early diagnosis and intervention. The objective of this study was to evaluate longitudinal records of patients with some early-diagnosed dental anomalies to estimate risks of developing PDC during the late mixed dentition.

## MATERIAL AND METHODS

This retrospective longitudinal study was approved by the Research Ethical Committee of the Hospital for Rehabilitation of Craniofacial Anomalies, University of São Paulo (HRAC-USP) (379/2010). The patient records were anonymized and de-identified prior to analysis. The initial sample was composed of the orthodontic files of 810 children treated from 1980 to 2005 at the Society for the Social Promotion of Cleft Lip and Palate Patients (PROFIS). Inclusion criteria were: presence of an initial panoramic radiograph taken during the first transitional period or inter-transitional period of mixed dentition, according to the Van der Linden^31^ (1983) classification, and presence of at least one more panoramic radiograph taken either during the second transitional period of mixed dentition or during the early permanent dentition. Exclusion criteria were: poor quality records (dark or distorted panoramic radiographs; absence of periapical radiographs in cases showing ectopic canines) and presence of syndromes or craniofacial anomalies. Eighty individuals were excluded based on these exclusion criteria.

The final sample was composed of 730 orthodontic records from children with an initial mean age of 8.3 years (SD=1.36), from both genders (448 females and 282 males). A rough estimate of the ethnic background of the sample based on facial photograph was: White (84%), Black (12%), and Asian (4%). The experimental and control groups included 263 and 467 records, respectively, and were composed based on the analyses of the initial panoramic radiographs and dental casts to investigate the presence of the following dental anomalies: 1. Agenesis of any permanent teeth, except for third molars; 2. Microdontia of maxillary lateral incisors; 3. Infraocclusion of deciduous molars; 4. Distoangulation of mandibular second premolars; 5. Tooth transpositions.

All the records were analyzed by a single calibrated examiner (ML). The examiner was precalibrated showing an agreement index ranging from 90 to 100% (Kappa test). The maxillary lateral incisor was considered as presenting microdontia when the maximum mesiodistal crown diameter was smaller than the same dimension of the opposing mandibular lateral incisor in the same patient, using the dental casts^19^. This category also included conical or peg-shaped maxillary lateral incisors. The presence of infraocclusion of deciduous molars was determined by visual inspection of the initial dental casts and panoramic radiograph series. A deciduous molar was considered in infraocclusion when more than 1 mm of vertical discrepancy was measured from the mesial marginal ridge of the closest permanent first molar^29^. Maxillary and mandibular first and second deciduous molars were considered in the analysis of infraocclusion. The diagnosis of distoangulation of mandibular second premolars followed the criteria described by Shalish, et al.^28^ (2002).

The sample was divided into two groups. The dental anomaly group (DA) was composed of 263 patients with at least one dental anomaly identified in the initial or middle mixed dentition. Records from children without these dental anomalies in the early/middle mixed dentition (n=467) composed the non-dental anomaly group (NDA). Age and gender distribution in both groups is presented in Table 1.

**Table 1:** Age and gender distribution in dental anomaly (DA) and non-dental anomaly (NDA) groups

	Mean age at first evaluation (SD)	Mean age at second evaluation (SD)	Male	Female
DA group (n=263)	8y2m (1.46)	10y10m (0.88)	95	168
NDA group (n=467)	8y6m (1.26)	10y4m (0.42)	187	280
Total (n=730)	8y4m (1.36)	10y8m (0.79)	282	448

Panoramic radiographs from late mixed dentition and/or early permanent dentition were evaluated to assess risks for the development of PDC in both groups. Considering the findings of Ericson and Kurol^15^ (1986) showing that the attempt to radiographically determine the eruption path of maxillary canines is generally of little value in children younger than 10 years old, we only examined panoramic radiographs in records from children aged 10 years or older. The PDC diagnosis followed the radiographic parameters suggested by Lindauer, et al.^22^ (1992) and was confirmed through the interpretation of periapical radiographs according to the Clark’s technique^12^. Rapid maxillary expansion (RME) performed during the mixed dentition was registered in both groups because RME may have a positive influence on PDC cases^6^.

The frequency of PDC development was calculated in DA and NDA groups. Intergroup comparisons were performed using the Chi-square test with a significance level of 5%. In order to measure the strength of associations between occurrences of early-diagnosed dental anomalies and PDC, the relative risk (RR) at the 95% confidence interval and the positive and negative predictive values (PPV and NPV) were calculated. Additionally, the frequency of PDC development was separately calculated for each dental anomaly and compared with the control group using the Chi-square test (p<0.01) and relative risk assessment.

## RESULTS

Seventy-two individuals were affected by PDC (9.86%) with a male:female ratio of 1:3 in the combined DA and NDA groups (n=730). In this subgroup of individuals with PDC, 29.1% (n=21) showed bilateral expression, 31.9% (n=23) unilateral right expression, and 38.9% (n=28) unilateral left expression.

The DA group presented PDC frequency of 16.3% compared with 6.2% of the NDA group (Table 2). This difference was statistically significant and indicated a two and a half fold increase in risk of PDC in patients with early-diagnosed dental anomaly (Table 2). Positive predictive value (PPV) corresponded to 16% and negative predictive value (NPV) was 93%.

**Table 2:** Comparison between dental anomaly (DA) and non-dental anomaly (NDA) groups based on the development of palatally displaced canines (PDC)

	DA group (n=263)	NDA group (n=467)	Pooled groups (n=730)	Difference chi-square (χ 2 )	Relative Risk (RR)	95% Confidence Interval
PDC Development	43 (16.34%)	29 (6.21%)	72 (9.86%)	18.33 p<0.01*	2.63	(1.69-4.11)
Absence of PDC	220 (83.65%)	438 (93.79%)	658 (90.13%)			
Total	263 (100%)	467 (100%)	730 (100%)			

* Statistically significant difference at p<0.01

Statistically significant associations were observed between increased frequencies of PDC development and some of the dental anomalies were separately evaluated (Table 3). The relative risk of PDC in children with these specific dental anomalies varied from 2.4 to 4.3 (Table 3). Tooth transposition was absent in the sample.

**Table 3:** Prevalences of palatally displaced canines (PDC) development associated with each separate dental anomaly compared with the non-dental anomaly group

Dental Anomaly	Number of patients	Prevalence of PDC Development	Prevalence of PDC in the NDA group	Difference chi-square (χ 2)	*p*	Relative Risk (RR)	Confidence interval (95%)
Mandibular second premolar distoangulation	49	26.53% (13/49)		21.85	<0.001*	4.27	(2.38-7.66)
Agenesis of mandibular second premolars	22	9.09% (2/22)		0.01	0.925	1.46	(0.37-5.75)
Agenesis of maxillary second premolars	8	25.00% (2/8)		1.99	0.158	4.03	(1.15-14.06)
Agenesis of maxillary lateral incisors	7	14.29% (1/7)	6.21% (29/467)	0.01	0.929	2.30	(0.36-14.61)
Small maxillary lateral incisor	82	23.17% (19/82)		23.07	<0.001*	3.73	(2.20-6.33)
Maxillary lateral incisor and/or second premolar agenesis	32	15.63% (5/32)		2.83	0.093	2.52	(1.04-6.06)
Deciduous molar infraocclusion	159	15.09% (24/159)		10.96	0.001*	2.43	(1.46-4.05)

* Statistically significant difference at p<0.01

The frequency of RME performed during the mixed dentition was similar in both DA and control groups (Table 4). No other type of transversal expansion was registered except RME. Extraoral traction was performed in 38.8% of DA group and 40.8% of NDA group. Serial extraction was performed in only one case of DA group and five cases of NDA group.

**Table 4:** Frequency of rapid maxillary expansion (RME) performed in both groups during mixed dentition and intergroup comparison (Chi-square test)

	RME	Non RME	Total	Difference chi-square (χ2)	*p*
DA group	144 (58.5%)	102 (41.5%)	246 (100%)	3.02	0.082
NDA group	219 (52.3%)	208 (48.7%)	427 (100%)		

## DISCUSSION

This study evaluated longitudinal records from patients with early-diagnosed dental anomalies to estimate risks for developing PDC during the late mixed dentition.

Previous cross-sectional studies showed an association between PDC and other dental anomalies including small maxillary lateral incisors, tooth agenesis, deciduous molar infraocclusion, and other slight tooth ectopia^2^ ^,^ ^19^ ^,^ ^25^. These studies evaluated the concomitant occurrence of canine ectopic eruption and other dental anomalies, and pointed to some risk indicators for PDC.

The present study is the first to evaluate a large sample with longitudinal records for dental anomalies that could be used as markers to estimate PDC risks^11^. Our results showed that children with early recognizable dental anomalies have an increased risk of 2.5 fold to develop PDC later in life compared with children without these anomalies (Table 2). According to positive predictive value (PPV), the frequency of positive results (presence of an early-diagnosed dental anomaly) that were true positive (patients who developed PDC) was 16%. Considering the negative predictive value (NPV), 93% of patients with negative results (absence of dental anomaly) were true negative and did not develop PDC. The results of this study corroborates that PDC belongs to a spectrum of interrelated dental anomalies^26^. The literature shows the occurrence of other dental anomalies concomitant with PDC^2^ ^,^ ^19^ ^,^ ^24^. Additionally, a higher prevalence of dental anomalies is observed not only in patients with PDC but also in their first and second-degree relatives^26^.

Small maxillary lateral incisors and mandibular second premolar distoangulation were the main risk factors for PDC among the early-diagnosed dental anomalies (Table 3). These results corroborate previous cross-sectional studies that demonstrated significant association between small maxillary lateral incisor and PDC^2^ ^,^ ^25^ ^,^ ^27^. Distoangulation of the mandibular second premolar was early described as a mild expression of the same genetic origin identified for antimere agenesis^28^. Recently, a cross-sectional study demonstrated a statistically significant difference between the prevalence of PDC (28%) in patients with distoangulation of the mandibular second premolars and in a control group (4.2%)^4^.

Deciduous molar infraocclusion was also confirmed to be a risk factor for PDC (Table 3); this association was previously reported in a cross-sectional study^29^. The prevalence of deciduous molar infraocclusion, reported from cross-sectional studies in a white population, varies from 1.3% to 8.9%^1^ ^,^ ^10^ ^,^ ^21^. The prevalence of deciduous molar infraocclusion in our combined sample (21.8%) was much higher than the frequency reported in previous studies (Table 3) and could be explained by our longitudinal period of observation.

No significant differences were observed between PDC development in individuals with agenesis of maxillary lateral incisors or second premolars, and the NDA group (Table 3). Peck, Peck and Kataja^25^ (1996) also showed that agenesis of maxillary lateral incisors was not significantly associated with PDC. On the other hand, previous cross-section studies have shown significant associations between PDC and second premolar agenesis^2^ ^,^ ^19^ ^,^ ^25^. Although second premolar agenesis has been previously identified as a risk indicator for PDC in cross-sectional studies, the association was not significant in our cohort evaluation; this difference could be explained by reference values, which considered general population frequencies in the former.

A limitation of this study is the possibility of false-positive diagnosis for PDC using panoramic radiographs^22^. However, the false-positive rate is low (4.22%) and seems not to compromise the study results^22^. The 80 individuals that were excluded from the sample were not analyzed either because the absence or bad quality of radiographs. Other limitation of this study could be the bias of sample selection because the study was retrospective. However, the sample selection followed the criteria of a time interval when the patients started the orthodontic treatment (from 1980 to 2005). Another concern regarding the methodology is that the early orthodontic treatment with RME might have had an influence on the spontaneous eruption of ectopic canines. However, both DA and control groups showed similar RME frequencies (Table 4) and the prevalence of PDC was still significantly higher in DA group.

Our results show that small maxillary lateral incisors, distoangulation of mandibular second premolar, and deciduous molar infraocclusion are early risk markers for PDC. Pediatric and orthodontic population with such dental anomalies diagnosed during the early mixed dentition should be carefully monitored during the critical age period for early diagnosis and intervention of maxillary canine ectopic eruption. The recognition of risk markers for the occurrence of PDC can increase the possibility of early diagnosis and intervention. Future longitudinal studies could contribute to identify other potential risk indicators for PDC including family history, female gender, hypodivergent pattern, and enamel hypoplasia^2^ ^,^ ^27^.

## CONCLUSION

Children with some dental anomalies diagnosed during the early mixed dentition have an approximately two and a half fold increase in risk of developing PDC during the late mixed dentition compared with children without these dental anomalies. Microdontia of maxillary lateral incisors, mandibular second premolars distoangulation, and deciduous molar infraocclusion constitute early risk markers for PDC development. When the maxillary canines are not palpable, a panoramic radiograph is highly recommended in 10-year-old children with clinically or radiographically diagnosed DA in order to investigate PDC.
